# An Exploration of Narcan as a Harm Reduction Strategy and User’s Attitudes toward Law Enforcement Involvement in Overdose Cases

**DOI:** 10.3390/ijerph19063149

**Published:** 2022-03-08

**Authors:** Jared Durieux, Andrew Curtis, Melissa Mirka, Eric Jefferis, Chaz Felix, Baaba Essel

**Affiliations:** 1 College of Public Health, Kent State University, Kent, OH 44242, USA; mmirka@kent.edu (M.M.); bessel@kent.edu (B.E.); 2 Department of Population and Quantitative Health Sciences, Case Western Reserve University, Cleveland, OH 44106, USA; ajc321@case.edu; 3 Tulare County Health and Human Services Agency, Visalia, CA 93277, USA; cwfelix@tularecounty.ca.gov

**Keywords:** homeless, naloxone, Narcan, spatial video geonarratives, skid row, police, harm reduction

## Abstract

The street homeless, those who spend their nights either in shelters or unofficial camps, whether in tents on a street or in society’s hidden spaces such as beneath an overpass, face multiple challenges beyond finding a safe place to sleep. Of further concern is how official actions can worsen these situations, through day-to-day activities or planned intervention strategies. In this paper we explore how a planned intervention may be negatively perceived—even as a form of “structural violence”—and may prevent Narcan (naloxone) use to stop an overdose related death in the Skid Row of Los Angeles. Data for this study consisted of a combination of Spatial Video Geonarratives (SVGs) and 325 incident reports from the Homeless Health Care Los Angeles Center for Harm Reduction (HHCLA-HRC) between November 2014 and December 2015. Chi-square and simple logistic regression models were used to examine the association between fear-of-arrest and other covariates of interest. Mapping results are presented with different sets of shapefiles created for (1) all Narcan uses, (2) all homeless, (3) all homeless with a worry about being arrested, (4) all Narcan uses where an ambulance attended, (5) and the same as 4 but also with police attendance. In the multivariable model, the estimated adjusted odds of fear-of-arrest is over three times higher among Narcan users ages 30–39 when compared to users under the age of 30. Analyzing the association of calling 9-1-1 on Narcan user demographics, socio-contextual characteristics, and overdose victim demographics, the crude estimated probability of calling 9-1-1 for Narcan users aged 50 and older is nearly three times higher when compared to Narcan users aged 19–29. Conclusion: Results suggest that the fear-of-arrest and calling 9-1-1 during an overdose is still a concern among Narcan users despite protective legislation and access to harm reduction resources.

## 1. Introduction

One of the tenets of *harm reduction theory* is that exposures resulting in dangerous or generally poor health situations is a function of individual behavior, choices, and also the settings and situations that frame those decisions [1]. A good example in the United States is the street homeless, and their high rates of substance abuse and overdoses. The street homeless, those who spend their nights either in shelters or unofficial camps, whether they be tents on a street or in society’s hidden spaces such as beneath an overpass, face multiple challenges beyond finding a safe place to sleep. Of further concern is how official actions can worsen these situations, through day-to-day activities or planned intervention strategies. In this paper we explore one such potentially negative planned intervention—a form of “structural violence”—preventing Narcan^®^ (naloxone) use to stop overdose related deaths in the Skid Row of Los Angeles. 

The street homeless are the most health compromised population in the United States. Among the diseases of concern are various forms of psychopathology, chronic disease, especially diabetes, and infectious diseases such as tuberculosis or sexually transmitted infections [2,3,4]. Additionally, the current COVID-19 pandemic has posed further health hazards to the homeless populations as these individuals are one of the categories that are most at risk for COVID-19 infections and are less likely to get the vaccine or to be tracked in order to minimize the spread of the virus [5]. Other exacerbating conditions include frequent exposure to interpersonal violence [6], a lack of access to health care, and concomitant substance abuse [7,8]. An ongoing challenge for health officials addressing these issues is the lack of data associated with homelessness. Aspects such as daily activity spaces, patterns of mobility, social and professional networks, and even the contact “home” address are largely unknown. Solutions to these data gaps can also be hampered by overzealous protection of this marginalized population by activists. While there is a risk of only discussing the homeless in terms of pathology due to the associated stigma it can create [9], it is also undeniable that illicit drugs are commonplace within many homeless communities [10,11]. There is therefore a need to provide contextualized data and analysis that can both inform public health departments, incorporate perspectives from the community at risk [12], and be practical enough to develop more sensitive interventions [13]. For example, if we are to reduce drug overdoses in the homeless community then we need to know not only where these occur, but why—*why* did this person use drugs and overdose, *why* at this location, and *why* were they not saved? 

The overdose situation in the United States as of 2021 is a major topic of concern, with frequent media stories re-stating this problem. Stories cover different geographic scales, from large cities to small towns, often highlighting various local nuances and attempted solutions. In the United States, mortality caused by drug overdose has been the leading cause of unintentional injury death since 2009, surpassing homicide and motor vehicle fatalities [14]. Moreover, overdose mortality has been associated with leading health indicators such as mental illness, poverty, and socioeconomic deprivation [15,16,17,18]. Among the homeless population, rates of drug use (and overdoses) are even worse. Research consistently shows over a third of individuals who are homeless experience alcohol and drug problems [19]. The relationship between homelessness and substance abuse is complex [20] and it is likely that the chance of an overdose in a street homeless setting is more likely to result in a death, partly due to the time it would take for traditional emergency services to attend and administer Narcan. Naloxone (Narcan) is a medicine that effectively reverses an opioid overdose [21]. One solution, again in keeping with harm reduction theory, is using the homeless as a resource, placing Narcan within the homeless community so that it can be quickly administered to those in need. This is exactly what the non-profit Homeless Health Care Los Angeles Center for Harm Reduction (HHCLA-HRC) has done in the Skid Row of Los Angeles. One problem, though, is for a homeless individual to become involved in such a reversal would mean handling a needle, which is seen by some as being an arrestable offense, and another problem is the willingness to interact with attending emergency services.

Los Angeles, the setting for this study, is recognized for accommodating the largest unsheltered homeless population in the United States. Of the 73,000 homeless, less than 17% live in shelters (i.e., congregate or private rooms). Compared to housed persons in the US, a homeless person’s life expectancy, on average, is 36% shorter. The largest concentration of homeless is approximately 50 square blocks located east of the Financial District and the Historic Downtown Center, an area commonly referred to as Skid Row. While reliable population counts are impossible, it is estimated that between 12,000 to 14,000 people live here, of which 75% are African Americans [22]. The importance of Skid Row to the homeless community extends beyond those who live there, as since the 1970s related services for the broader city have moved here [23]. For example, one study found 70 not-for-profit groups and approximately 3300 beds [24] in the area.

### 1.1. Spatial Video Geonarratives

According to a report on the LA Coroner’s Office statistics by the National Coalition for the homeless, the second most common cause of death in this cohort was acute intoxication from drug (heroin and morphine) or alcohol misuse [25]. The complexity of neighborhood drug situations has been discussed in previous research by some of the authors of this paper, who have produced sub-neighborhood scale contextualized maps of substance use using spatial video geonarratives (SVG) [26,27]. SVG is an environmentally inspired interview, with spatially encoded video being recorded along with commentary, so that important spaces, and their meaning and/or interpretation, can be mapped and analyzed together. This approach has been successfully used to capture drug and marginalized landscapes in a variety of different locations across the United States, but especially in Skid Row, Los Angeles [26,27]. SVG not only generates data to map objects (such as camp locations) but also provides the social setting of these places. As a result, different drug geographies of Skid Row were identified, such as where heroin dominates over cocaine, where there were hotspots for overdoses, and what were the local community responses to those overdoses. These SVGs not only reveal the negative, but also showcase many examples of positive community interaction. As was stated earlier in the paper, many homeless camps are thriving positive spaces, full of caring “friends”, and should be considered a resource. Indeed, “community as a resource” was a common thread through many of the SVGs, with Skid Row being divided into many such gatherings, sometimes as small as a cluster of tents where the occupants knew each other and looked out for each other.

*“I mean they kind of look out for each other. So you’ve got for example on San Pedro, there was like there was all those tents on the West side of the street and this one woman told us that they were worried about this other woman in another tent but she wasn’t there when we got there, this was yesterday and so the women were in these tents, were trying to take care of her so you definitely have like these little mini communities.”*

Not only did these gatherings provide a social fabric and network, but they also provided the means for a first response to an overdose. This was not lost on Skid Row providers who commented that the only effective interventions, such as health education, had to come from within. Not only was it a convenience issue, but one of trust and communication. 

The SVGs also provided an insight into the more troubling role that officialdom could have in exacerbation of drug use and overdoses. For example, one subject explained how after receiving drug treatment counseling after an overdose, he then felt “punished” as he was released back into the same conditions as before. To address this type of despair, the (for some divisive) “housing first’” approach focuses on finding stable living before addressing substance abuse [28]. The rationale being unless the individual can improve their living situation more holistically, then there is not a lot of point focusing on only one aspect [22]. The role of policing frequently emerged as well. For example, consider the following quote from a provider SVG.

*“[…] we got needle exchange authorized legally […] but the last lieutenant actually came in to talk to us and I showed him the legal statutes and I was like you know this is where it says like you can possess syringes, it’s not illegal anymore and he said honestly I don’t care because in my eyes it’s still a crime so I’m going to tell them to arrest those people […] I don’t care, I think it’s a crime.”*

Structural violence with regards to street homelessness is not new [29]. Other examples have included “red lining” through criminalization, which means banning individuals from a certain area if they “break the law”, which can have the effect of placing a barrier between the homeless and various services [11]. The criminalization of the homelessness can also justify street sweeps, which is a means to target drugs of all types, resulting in spending the night in jail [25,30]. Unfortunately, both actions can have negative health implications, and even cause death. Locking a drug user up overnight, and especially over the weekend, can reduce tolerance and lead to an overdose. Street sweeps also remove legal medication needed by a cohort suffering from multiple different chronic illnesses, and also Narcan. Having the homeless hold Narcan fits with the narrative of making this community a resource in themselves, to be their own first responders, and reduce the number of overdose-related deaths. For example, consider the following quote:

*“One of the other buildings and um, a girl was overdosing and they pulled her into one of the hotels and he was saying ‘hey, hey hold on a second’ he goes ‘I can get Narcan, I can save her’ and he’s like ‘call 911’ and they’re like ‘man, we ain’t calling 911′ he runs to the exchange bangs on the door, we’re about to close, bangs on the door, like ‘GIVE ME NARCAN, GIVE ME NARCAN!’ We give him Narcan, he goes back, he looks for the girl, because they had her inside one of the hotels, he’s like screaming at people and was actually able to give her Narcan.”*

In this paper we attempt to explore the idea of, to what degree is the fear of the police associated with the likelihood of overdoses in the Los Angeles Skid Row? To do this we analyze Narcan surveys collected by HHCLA-HRC whenever a renewal is requested. More specifically, we investigate the reported fear of arrest associated when a “good Samaritan” uses Narcan to prevent an overdose mortality and ponder to what degree is this “fear” resulting in preventable deaths. 

### 1.2. Methods

#### 1.2.1. Sampling

Secondary data consisting of incident reports from the Homeless Health Care Los Angeles Center for Harm Reduction (HHCLA-HRC) between November 2014 and December 2015 were acquired in paper form and digitized by the researchers. HHCLA-HRC is a community-based organization that provides preventative health interventions and medical services, including Narcan distribution to all homeless, though because of its location in Skid Row, most frequently to those living in that neighborhood. For someone to receive a new dose of Narcan, an incident report must be completed by trained HHCLA-HRC staff. A total of 325 incident reports were provided by Narcan users in response to overdose incidents and used for the analyses of this paper. To check for accuracy against the original documents, the digitized records had a random 10% sample checked against the paper forms by a second researcher. 

#### 1.2.2. Measures

The incident report contains a total of 27 items, including both the respondents’ and overdose victim’s socio-demographics, the overdose setting, method of administration, overdose response including police involvement in the overdose event. Covariates of interest for this study included: age, gender, ethnicity, homelessness, relationship to overdose victim, Narcan user’s response to the overdose, first responders and police involvement, and fear-of-arrest. Questions on the report varied in terms of responses; some were binary (was the Narcan user homeless), while others had up to 12 possible response options. The incident report also contained several spatial variables, including where the person administering the Narcan (if homeless) slept the previous night, and where the overdose incident occurred. 

#### 1.2.3. Data Analysis

Geopy, a python-based library for geocoding, which supports Google geocoding services, was used to convert the combination of street number, street intersection, and neighborhood to coordinates. Although the incident reports contained spatial data that extended across all of Los Angeles County, this area was set as a boundary to improve geocoding accuracy. Google geocoding services returned coordinates of type point of interest (poi), political regions, route (roadways), intersection, postal code, and street address. For this study only, selected coordinates that have coordinate type as point of interest (poi) such as a shelter name, an intersection, or street address, were used. 

Incident reports were mined for the socio-demographics of the person applying the Narcan and that of the overdose victim’s demographics on the likelihood of a Narcan user to call 9-1-1. Highly correlated variables, such as first responders and police involvement, were any arrests witnessed, or if the victim survived, were not included in this part of the analysis. All univariate and multivariable analyses were restricted to complete cases. Descriptive statistics were computed for the covariates of interest and the overdose circumstances including the drug(s) used and the method of administration in addition to actions taken in response to the witnessed overdose. Chi square was used for all bivariate analyses due to the variables all being nominal in nature, with the exception of age which was coded in an ordinal fashion. The dependent variables, “fear of arrest” and “called 9-1-1” were dichotomous in nature. The association between fear-of-arrest and covariates of interest were examined using chi-square and simple logistic regression. To describe significant relationships, unadjusted odds ratios, adjusted odds ratios and corresponding 95% confidence intervals (CI) were computed. Differences between groups were considered statistically significant at α < 0.05. All analyses were performed in SAS 9.3 (SAS Institute, Cary, NC, USA).

## 2. Results

A total of 325 incident reports were used in this analysis. The typical Narcan user was 50 or older (28.09%), male (72.31%), and white (48.61%). Among Narcan users, significantly more were over 50 years old, 28.09% (*n* = 75; chi square 12.29, *p* = 0.006), and 48.61% (*n* = 157; chi square 11.92, *p* = 0.007) were white, when compared to the overdose victims. Females comprised 28.00% (*n* = 90) of Narcan users (Table 1).

For the purposes of mapping, as the incident reports contained data from across Los Angeles County, we constrained responses to Skid Row to coincide with the SVG data through a clip operation. Different sets of shapefiles were created for (1) all Narcan uses, (2) all homeless, (3) all homeless with a worry about being arrested, (4) all Narcan uses where an ambulance attended, (5) and the same as 4 but also with police attendance. Kernel density estimate plots were generated for each data set with a fixed cell size of 4.5 m and best bandwidth option available through ESRI ArcGIS tool. 

**Figure 1:**
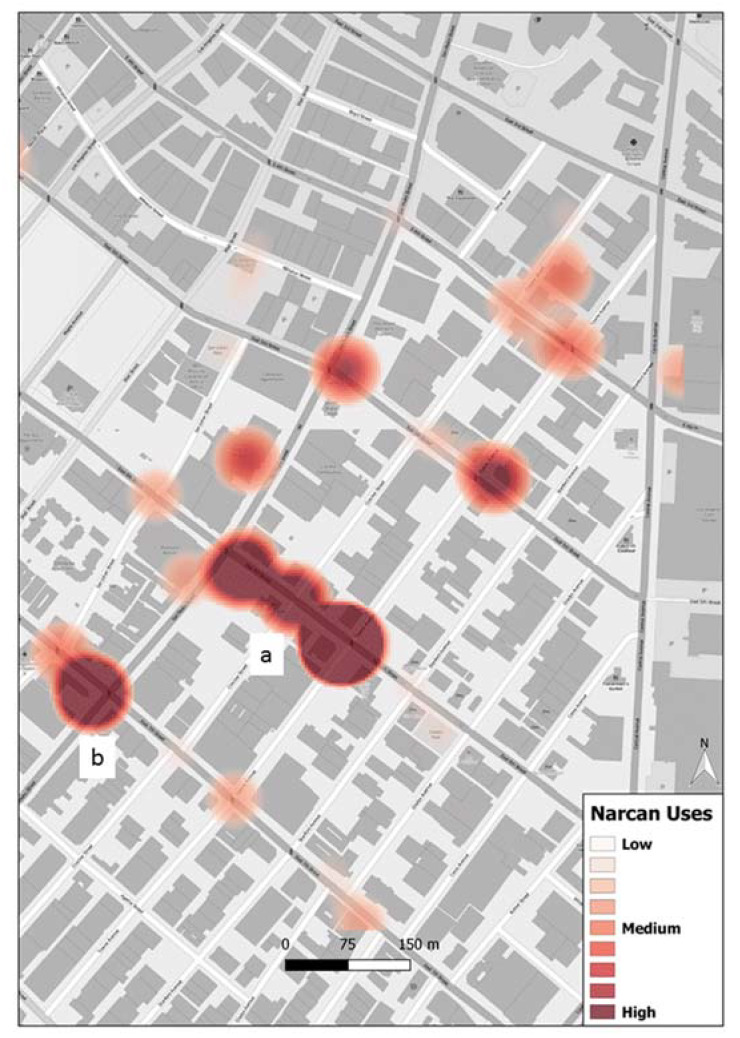
The locations of all Narcan applications during the study period in the Skid Row of Los Angeles. Areas a and b indicate two primary hotspots of Narcan applications.

**Figure 2:**
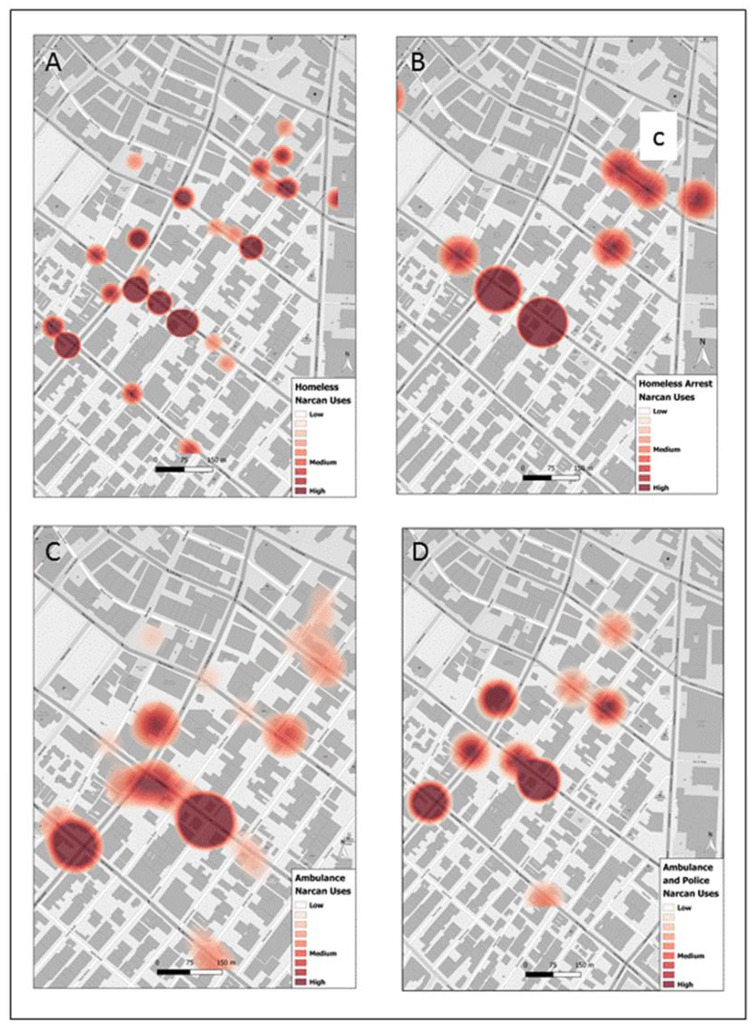
The locations of all Narcan reversals administered by a self-reporting homeless individual (**A**) who also expressed a worry about being arrested (hotspot area c) (**B**), where an ambulance attended the reported overdose (**C**) and lastly where the police also attended along with the ambulance (**D**).

Applications of Narcan within Skid Row are clustered with two main hotspots (a and b) and several smaller concentrations (Figure 1). When focusing on just the homeless holding Narcan, while the same two hotspots a and b remain, there is a more widespread coverage, suggesting a cohort more aligned with where overdoses are likely to occur (Figure 2). Interestingly, it is the fear of arrest map that again reemphasizes the location a, though another area c also emerges as roads where there is concern of arrest. 

The number of Narcan users who called 911 and feared arrest, as well as socio-contextual characteristics of Narcan uses, are presented in Table 2. Sixty-five (21.31%) Narcan users indicated fear-of-arrest if 9-1-1 was called.

For socio-contextual characteristics of Narcan users, 76% (*n* = 233) of respondents were either a sex partner, friend, relative, or an associate of the overdose victim. Among opioid analgesics, the primary method of administration was injection in a vein, 89.51% (*n* = 290). A third of the respondents (*n* = 110) reported calling 9-1-1 to an overdose event and out of those number of calls, the police arrived in 48 of these events (32.88%). Although 21.31% (*n* = 65) of Narcan users feared arrest by police, only 3 arrests were reported. The ambulance arrived in about two-thirds of overdose events (*n* = 116) and 96% (*n* = 306) of overdose victims survived.

Multivariable models with fear-of-arrest and call 911 as the dependent variables are shown in Table 3. These include self-reported demographics of Narcan user, overdose victim, and socio-contextual characteristics by fear-of-arrest (Model 1 Outcome) and by called 9-1-1 (Model 2 Outcome). Relationships were considered significant at α < 0.05.

In the adjusted models, female Narcan users were 39% less likely to fear arrest when the police arrived at an overdose event [OR; 0.39 (0.15–0.89)]. Holding all explanatory variables constant, the odds of fear-of-arrest was over two times higher among Narcan users aged 30–39 when compared to Narcan users under the age of 30 [OR; 3.42 (1.23–10.68)]. When analyzing the association of calling 9-1-1 on Narcan user demographics, the estimated adjusted odds of calling 9-1-1 is almost twice among Narcan users aged 50 and older when compared to Narcan users aged 19–29 [OR; 2.70 (1.11–6.92)]. Holding all explanatory variables constant, non-homeless Narcan users are 36% less likely to call 9-1-1 compared to homeless Narcan users [OR; 0.36(0.16–0.75)]

Analyzing the association of calling 9-1-1 and fear of arrest on Narcan user demographics and their socio-contextual characteristics, the unadjusted odds results showed that Latino Narcan users are 1.25 times more likely to call 9-1-1 compared to White Narcan users [OR; 2.25 (1.27–3.99)]. In addition, Latino Narcan users were 44% less likely to fear arrest when the police arrived at an overdose event. [OR; 0.44 (0.19–0.93)].

## 3. Discussion 

Gatherings of the homeless pose considerable problems in terms of adequate cultural and socially appropriate health care delivery. This is a complex situation not helped by public health teams being data-poor. To address one aspect of this, for one location, the Skid Row of Los Angeles, we analyzed a non-traditional dataset for insights into drug overdose situations. The paper more specifically considers how a program that utilizes the homeless as a resource, and places Narcan *on the street*, suffers because of the perceived “structural violence” due to a fear of police involvement. This was explored using 325 overdose incident reports from the Homeless Health Care Los Angeles Center for Harm Reduction (HHCLA-HRC) between November 2014 and December 2015. While this study was exploratory in scope, the analyses revealed several noteworthy findings. In terms of the demographics of the study, just over 23% of those requesting Narcan were African American—yet previously mentioned research suggests that the homeless population of Skid Row is 75% African American [22]. This is obviously a huge difference which leads to questions of, why, perhaps, are more African Americans less willing to hold Narcan? For example, is it a question of not being aware of the opportunity, or a fear of arrest? Secondly, to what degree does this racial imbalance lead to a greater likelihood of an African American overdose resulting in a death?

We further analyzed these data for examples of structural violence, especially with regards to police involvement. First, when modeling fear-of-arrest as a function of Narcan user demographics, socio-contextual characteristics, and overdose victim demographics, we identified three factors of fear-of-arrest: age, gender, and ethnicity of Narcan user. Among Narcan users, we estimated that fear-of-arrest was significantly higher among respondents aged 30–39 but lower among female and Latino Narcan users. At this time, study participants observed that the police arrived on scene with the ambulance in less than half (41.38%) of events during which 911 was called. Of those 48 instances, only three arrests were made. This is the same frequency of arrest (*n* = 3) as previously reported [31], yet the prevalence of police response is lower in our study.

Comparing the UOR to the AOR in model 1, the crude estimate for Narcan users ages 30–39 was not a significant factor in fear-of-arrest. When estimating the adjusted odds ratios, for Narcan users aged 30–39 the fear of arrest becomes a significant factor.

Second, after we analyzed the effect of demographic and socio-contextual variables on the likelihood of Narcan users to call 9-1-1, three explanatory variables were identified as independent predictors of calling 9-1-1: age, ethnicity, and housing status of Narcan user. We estimated that Narcan users who were 50 years old and older or Latino were more likely to call 9-1-1 and non-homeless Narcan users were less likely to call 9-1-1 in response to an opiate overdose. In this model, neither gender nor Narcan user’s relationship with the victim were factors of calling 9-1-1. 

Comparing the UOR to the AOR in model 2, the crude estimate for Latino Narcan users was significant. When estimating the adjusted odds ratios, the estimated effect of Latino Narcan users to call 9-1-1 was no longer significant. The changes in the association between Latino Narcan users and call 9-1-1 suggest a confounding relationship. However, in model 1 and model 2, the differences in the estimated effects between the unadjusted and adjusted odds was moderate-to-nominal, which implies that the extent of confounding was minimal.

These discoveries indicate that gender has a differentiating effect. Our earlier findings stated that the proportion of females who called 9-1-1 was larger than males. Likewise, a previous study stated that when female Narcan user were at the scene of an overdose, 9-1-1 was more likely to be called [32]. Although gender was not a significant factor to call 9-1-1 in our sample, the direction of the relationship suggests females were less likely to fear arrest than males. This may not fully explain the variation in fear-of-arrest, however, these results do provide insight into the practical differences between males and females in this population. 

It has been suggested that previous history with law enforcement [32], lack of protective legislation [31], increased policing [33] or threat of incarceration [34] are barriers to calling 9-1-1 when witnessing a drug overdose. Although we were not able to answer each of these inquires with the available data, our analysis indicated that in a predominantly homeless population, that age, gender, and ethnicity of Narcan users and ethnicity of overdose victim are factors of fear-of-arrest, and that the effects of age, ethnicity, and housing status of Narcan users are factors of calling 9-1-1 in response to an opiate overdose. 

To the best of our knowledge, there is only one study to date that analyzes incident reports from HHCLA-HRC [28]. Our study, however, is the first to examine Narcan users’ attitudes towards law enforcement involvement in overdose occurrences. Since the publication of that study [28], decriminalization legislation that encourages Narcan users to call 9-1-1 in drug overdose situations has been passed [35]. The current study found that the fear-of-arrest is still a concern among Narcan users despite protective resources such as the Good Samaritan Law and access to mobile technology.

Future research should investigate why males are more likely to fear arrest than females. Is it because they are dealing or is it because of previous encounters with law enforcement? Likewise, additional studies are needed to investigate the correlates that effect non-White ethnic groups. For example, why are Latino Narcan users less likely to fear arrest and more likely to call 9-1-1? Additionally, future studies should consider the lived experience of Narcan users. This might provide valuable insights into understanding what it is like to make the decision to administer Narcan. 

## 4. Limitations

The limitations of this study were those typically associated with secondary analysis.

1. First, the incident reports provided to us were not created with our study in mind but for a different purpose. This adds a level of difficulty in defining and operationalizing variables.
2. The data used for these analyses are cross-sectional, eliminating the ability to make causal inferences.
3. Third, the variables we selected for our study were restricted by the nature and structure of questions available on the incident report. This minimizes our ability to test certain hypotheses.
4. Fourth, since response items were filled in by staff hand, there is always the possibility for missing or incomplete data and inconsistencies in how the forms were completed and stored. At this level, errors such as these may have contributed to response bias or socially desirable reporting. Since we did not impose balance on the sample by estimating and imputing missing values, the possibility of significance bias or reduced precision of estimates should be noted.
5. Fifth, although our unit of analysis was the incident and not the individual, the unique identifiers used on these forms made it difficult to discern if the observations were independent of each other or if there were the possibility of multiple records per an individual. The discrepancy between the proportion of African American Narcan users in our sample (23 %) compared to previous research (75%) [15] in Skid Row may have various explanations (e.g., sampling, varied fear of police), but cannot be explained by these data and must be further explored.
6. Sixth, recall bias is also a concern. It is unknown how much time passed between the witnessed overdose and completion of the survey. Depending on the amount of time lapsed, some events and overdose circumstances may be difficult to recall. In addition, and as stated in this quote from the SVGs, there was also a reluctance for some to give information:
  *“So we do complete self-reporting, so if someone comes to us and they say “I used my kit”, we try and get as much information as possible, but they don’t want to disclose it.”*
7. Lastly, as our questions grew in sophistication, many categorical variables had to be collapsed or removed from the analysis due to missing data or the small number of observations that fit the criteria of our inquiry.

## 5. Conclusions

Fear-of-arrest is still a concern among Narcan users in spite of protective legislation and access to harm-reduction resources. Our findings suggest that, independent of ethnicity, housing status of Narcan users, and relationship with the overdose victim, the influence of the overdose victim’s age and gender were factors of fear-of-arrest. Similarly, age, ethnicity, and housing status of Narcan users were factors of calling 9-1-1 in response to an overdose event. Even when Narcan users were stratified by calling 9-1-1, ethnicity was a cause for fear-of-arrest.

Our results add to the growing body of literature on the factors that impact Narcan users’ fear-of-arrest in overdose events. Similar to the challenges faced by the homeless population in relation to vaccine acceptance, these findings might inform the public health community of the challenges the homeless population face resulting in mistrust in government agencies that increase their health hazards [36]. Understanding the underlying causes that influence behavior provides evidence that can influence legislation and public programs that focus on prevention, harm-reduction, and treatment. Reducing fear-of-arrest among Narcan users increases the likelihood of achieving a health-centered approach to drug misuse. 

## Figures and Tables

**Table 1:** Demographics of Narcan user and overdose victim.

	Narcan User		Overdose Victim	
	*n*	%	*n*	%
Age *				
19–29	52	19.48	91	30.85
30–39	73	27.34	79	26.78
40–49	67	25.09	69	23.39
50+	75	28.09	56	18.98
Gender				
Male	235	72.31	229	72.47
Female	90	27.69	87	27.53
Ethnicity *				
White	157	48.61	137	44.34
Hispanic/Latino	79	24.46	52	16.83
Black/African American	76	23.53	106	34.30
Asian/American Indian/Mixed/Other	11	3.41	14	4.43

Demographics (Age groups, Gender, and Ethnicity) of Narcan User and Overdose Victim. Total sample size 325. Note: * indicates significance at the 0.05 level using chi square test.

**Table 2:** Narcan users’ socio-contextual characteristics.

	N (*n* = 325)	%
Housing status of Narcan user		
Homeless	245	75.38
Not homeless	80	24.62
Relationship with Overdose Victim		
Association (sex partner, friend, relative, associate)	233	76.14
No association (stranger)	73	23.86
Drug Misused		
Heroin	309	95.37
Speedball	9	2.78
Other opiates	7	2.16
Methadone	1	0.31
Method of Drug Administration		
Injection in a vein	290	89.51
Called 9-1-1		
Yes	110	33.95
No	214	66.05
Victim Survived		
Yes	306	95.92
No	13	4.08
Fear-of-Arrest		
Yes	65	21.31
No	240	78.69
Stayed with Victim		
Yes	266	88.37
No	35	11.63
	**N (*n* = 116)**	**%**
If 911 called, Ambulance Arrived		
Yes	116	100.00
If 911 called, Police Arrived with ambulance		
Yes	48	41.38
If 911 called, Arrest made		
Yes	3	6.67
No	42	93.33

Narcan users’ socio-contextual characteristics includes relationship of Narcan user with overdose victim, reported drug misused, the method of drug administration, if the victim survived, and frequency of arrests.

**Table 3:** Estimated unadjusted (UOR) and adjusted (AOR) odds ratios and 95% confidence interval (CI) by fear-of-arrest and call 9-1-1.

	Fear-of-Arrest		Call 9-1-1	
	UOR (95%CI)	AOR (95%CI)	UOR (95%CI)	AOR (95%CI)
Narcan User Demographics				
Age				
19–29	1.00	1.00	1.00	1.00
30–39	1.89 (0.77–5.01)	**3.42 (1.23–10.68)**	0.86 (0.37–2.01)	0.94 (0.38–2.37)
40–49	0.96 (0.35–2.70)	1.60 (0.52–5.34)	0.94 (0.41–2.21)	1.17(0.46–2.94)
50+	0.96 (0.36–2.68)	2.21 (0.70–7.57)	2.92 (1.37–6.50)	**2.70 (1.11–6.92)**
Gender				
Male	1.00	1.00	1.00	1.00
Female	**0.46 (0.21–0.92)**	**0.39 (0.15–0.89)**	1.44 (0.86–2.39)	1.08 (0.56–2.07)
Ethnicity				
White	1.00	1.00	1.00	1.00
Hispanic/Latino	**0.44 (0.19–0.93)**	**0.33 (0.11–0.86)**	**2.25 (1.27–3.99)**	1.63 (0.78–3.38)
Black/African American	0.78 (0.39–1.49)	0.54 (0.22–1.25)	1.10 (0.61–1.96)	1.13 (0.54–2.33)
Asian/American Indian	0.36 (0.02–2.05)	0.51 (0.03–3.62)	0.59 (0.09–2.48)	0.79 (0.11–3.90)
Socio-contextual Characteristics				
Housing status of Narcan user				
Homeless	1.00	1.00	1.00	1.00
Not homeless	0.83 (0.42–1.58)	0.62 (0.26–1.37)	0.36 (0.19–0.65)	**0.36 (0.16–0.75)**
Relationship with Overdose Victim				
Association (sex partner, friend, relative, associate)	1.00	1.00	1.00	1.00
No association (stranger)	0.67 (0.34–1.27)	0.66 (0.28–1.48)	1.04 (0.62–1.7)	0.98 (0.51–1.86)

The underlined text illustrates the nature of the variables presented below. Values in bold font indicate significant odds ratios.

## Data Availability

Data requests can be made through the corresponding authors.
